# 基于核酸适配体信号置换结合循环扩增的液相色谱法检测4种生物胺

**DOI:** 10.3724/SP.J.1123.2022.07004

**Published:** 2022-11-08

**Authors:** Chang SONG, Chang LIU, Ziyu MA, Ruirong PAN, Haiwei SHI, Dezhao KONG, Jinghui ZHANG, Wei SHEN, Sheng TANG

**Affiliations:** 1.江苏科技大学环境与化学工程学院, 江苏 镇江 212003; 1. School of Environmental and Chemical Engineering, Jiangsu University of Science and Technology, Zhenjiang 212003, China; 2.江苏科技大学粮食学院, 江苏 镇江 212003; 2. School of Grain Science and Technology, Jiangsu University of Science and Technology, Zhenjiang 212003, China; 3.江苏大学附属医院, 江苏 镇江 212001; 3. Affiliated Hospital of Jiangsu University, Zhenjiang 212001, China; 4.江苏省食品药品监督检验研究院, 江苏 南京 210019; 4. Jiangsu Institute for Food and Drug Control, Nanjing 210019, China

**Keywords:** 液相色谱, 核酸扩增策略, 生物胺, 抗生素, 鱼肉, 猪肉, liquid chromatography (LC), nucleic acid amplification strategy, biogenic amine, antibiotics, fish, pork

## Abstract

生物胺的含量是衡量食品卫生状况和药物纯度的重要标志之一,建立食品药品中生物胺的精准、灵敏检测具有重要的实际意义。该文基于核酸适配体置换生物胺信号源并结合荧光信号循环扩增的策略,建立了一种新型的同时检测鱼肉、猪肉和抗生素中4种生物胺的高效液相色谱法(HPLC)。首先通过两步信号置换,将无荧光信号的目标物转换为有荧光信号的核酸探针;再结合双链特异性核酸酶辅助信号扩增策略,获取大量不同长度和碱基序列的核酸探针;最后借助HPLC平台实现实际样品中多种生物胺信号的精确识别。文章研究了核酸探针的碱基序列和长度对出峰时间和前后顺序的影响,以提高荧光信号的区分度。通过正交实验探讨了柱温、流速和梯度洗脱过程、反应温度、孵化时间等对信号分离的影响,确定最优条件,提高信号的分离效率。该方法对目标物酪胺、组胺、精胺和色胺的检出限分别为0.25、0.21、0.27和0.19 pmol/L,线性范围为1 pmol/L~1 μmol/L。通过对硫酸大庆霉素、鱼肉和猪肉样品中生物胺含量进行检测,研究了该方法检测实际样品的可行性。该方法可精准识别、捕获和分离复杂基质样品中的生物胺组分,能有效提高对目标分析物的选择性,并降低实际样品中的基质干扰,有望为食品药品分析领域提供一种新的思路。

## Abstract (en)

Biogenic amines (BAs) represent a class of potentially harmful substances in foods and medicines. Their content is thus an important indicator of proper hygiene in food preparation, and purity of medicines. It is of great practical significance to establish accurate and sensitive detection of BAs in food and drugs. In this study, a high performance liquid chromatography (HPLC) method was developed for the simultaneous detection of multiple BAs in fish, pork and antibiotics based on aptamer signal replacement and cyclic amplification strategy. First, non-fluorescent targets were converted into fluorescent nucleic acid probes using a two-step replacement process. Subsequently, a large number of nucleic acid probes with different lengths and base sequences were generated using a double-stranded specific nuclease-assisted signal amplification strategy. Finally, various BAs in real samples were accurately identified using an HPLC platform. The influence of base sequence and nucleic acid probe length on separation via HPLC was studied to improve discrimination among fluorescent signals. Four different sequences were selected as tails to the DNA probe, and their retention times increased in turn. Experimental conditions, including column temperature, flow rate, gradient elution process, reaction temperature, and incubation time, were optimized by orthogonal experiments to further improve signal separation efficiency. Specifically, the methanol gradient was changed from 10% to 20% during 0-20 min, 35 ℃ of column temperature and 1.0 mL/min of flow rate were chosen as the HPLC conditions. The final resolution of chromatographic peaks was 3.44, 3.59 and 2.37, indicating complete separation between peaks. Optimal incubation time for BA capture by aptamer was 120 min, and optimal dosage of duplex specific nuclease (DSN) and Mg^2+^were 0.9 U and 30 mmol/L. The optimal pH, incubation temperature, and DSN incubation time were 7.0, 40 ℃ and 210 min, respectively. The proposed method exhibited high sensitivity towards BAs, with a linear range of 1 pmol/L-1 μmol/L, and the limits of detection of tyramine, histamine, spermine, and tryptamine were 0.25, 0.21, 0.27 and 0.19 pmol/L, respectively. The feasibility of this method was verified, and contrast experiments indicated that it could achieve highly selective detection of four BAs in one run. The applicability of this integrated method was also investigated for the detection of real samples (gentamycin sulfate, fish and pork). To assess the matrix effect, each BA with different concentrations were spiked into real fish and pork samples. Relative recoveries and relative standard deviations (RSDs) ranged from 101.2% to 104.5% and from 1.5% to 4.3%, respectively. The above detection results for real samples showed that this method could accurately capture, separate, and identify BAs in complex matrix samples. This strategy can effectively improve analyte selectivity and reduce the matrix effect. This assay is thus expected to provide a new approach for food and drug analyses.

生物胺是一类有生物活性的、含氮的、相对分子质量较低的有机物质的统称^[1]^。按照生物胺的化学构成可以将将其分为腐胺、尸胺、精胺(spermine)、亚精胺等脂肪族胺,酪胺(tyramine)、苯乙胺等芳香族胺以及组胺(histamine)、色胺(tryptamine)等杂环胺^[2,3]^。低水平的生物胺是生物蛋白质中至关重要的部分,在调控核酸与蛋白质的化学组成及生物膜稳定性等方面起着重要作用^[4]^。然而一些高水平的生物胺则被归类为有害化合物,可能会在敏感的人体中引起疾病或不良症状^[5]^。生物胺的高摄取量(食物中超过100 mg/kg)可能对人类健康构成威胁^[6]^。组胺是所有生物胺中毒性最强的,过量的组胺会导致头痛、消化功能障碍及血压失常,甚至还可能产生神经性中毒^[7]^。酪胺的毒性次之,过量可能会导致恶心和高血压等不良反应^[8]^。尸胺和腐胺本身危害性相对较小,但却能抑制体内组胺和酪胺相关代谢酶的活性,促使组胺和酪胺的过量积累,加剧身体的不适症状^[9]^。除此之外,腐胺、尸胺、精胺和亚精胺能够与亚硝酸盐反应,生成致癌产物亚硝基胺。生物胺主要的外部摄入来源是部分常见的食品或药品,如抗生素和肉制品等,近年来开始受到各国食药监管部门的重视。大部分抗生素由微生物发酵制得,然而此方法具有产物纯度较低、工艺可控性较低、生产稳定性差、活性组分易变异等问题。发酵生产过程中所产生的生物胺杂质可能引起中毒等一系列不良反应^[10]^,如研究发现发酵生成的庆大霉素中的组胺浓度与不良反应事件之间存在明显的相关性。鱼肉和猪肉中含有丰富且极易被降解的蛋白质,再加上肉类制品有利于细菌生长繁衍,各种微生物的大量繁殖很容易导致鱼肉和猪肉中生物胺浓度超标并且种类繁杂。如在鱼肉中可以检测到组胺、酪胺,在猪肉中常可以检测到组胺、精胺和色胺。因此,精确检测食品药品中生物胺(尤其是组胺、酪胺、精胺和色胺)含量是否超标具有重要意义。

传统的检测生物胺的策略主要包括色谱法、光谱法、电泳法、比色法、化学发光法和电化学方法等^[11]^。其中常用的色谱方法有薄层色谱法、离子交换色谱法、气相色谱法、高效液相色谱法(HPLC)等,并结合不同的检测器进行检测^[12,13]^。然而,传统的色谱法检测多种生物胺不仅需要复杂的衍生化步骤以及繁琐的样品前处理程序,而且有时无法在一次运行中检测多种不同种类的生物胺,需要选择不同的色谱方法对同一个样品进行多次检测。同时,常规的色谱检测方法无法有效解决抗生素中混合物基质干扰的难题,在目标生物胺含量低的时候,目标峰常常淹没于杂质谱中,无法有效识别。此外,较窄的线性范围和较高的检出限(LOD)也是限制传统色谱法准确识别多种生物胺的主要因素。

核酸适配体(aptamer)作为一种新型的检测探针,被广泛应用于各种检测分析技术之中^[14,15]^。核酸适配体是一段寡核苷酸序列,能与特定的目标物质高特异性、高选择性结合。目前,针对生物胺的适配体也已被广泛筛选并应用于检测方法之中,如Duan等^[16]^、Tian等^[17]^、John Ho等^[18]^通过指数富集法分别筛选出了酪胺、精胺和组胺的适配体。核酸适配体对生物胺的选择性结合和分离能够有效排除基质效应的影响,从而提高检测的选择性和灵敏度。

信号扩增方法能进一步增强检测的灵敏度,已成为检测痕量目标物的一种有效手段。在之前的工作中,Qi等^[19]^开发了一种基于双链特异性核酸酶(DSN)辅助增敏策略用于多种微RNA(microRNA)检测的HPLC方法。该方案通过DSN剪切过程,循环放大来自microRNA的信号,并结合剪切的长短核酸探针实现了多种microRNA在HPLC上的信号分离,有效进行了目标信号源的置换工作;同时结合色谱法实现了对不同长度核酸探针的同时检测,实现目标物质的多重检测。因此,若能将适配体检测策略与信号扩增方法相结合,再借助高灵敏的HPLC平台,则有望实现在一次运行中对多种生物胺进行快速识别和检测。

基于此,本文提出了一种基于适配体置换生物胺信号源并结合荧光信号循环扩增技术的HPLC方法,并将其应用于抗生素和肉类中多种生物胺的识别和检测。该方法的优势在于:(1)通过启动子(trigger)置换出生物胺信号,再由核酸探针置换出启动子信号。两步信号置换可将分析物从无荧光信号的生物胺转换为有荧光信号的核酸探针,从而精准识别、捕获和分离硫酸庆大霉素、鱼肉、猪肉复杂基质中的生物胺成分,提高对分析物的选择性,降低样品的基质干扰;(2)RNA启动子(RNA不会被DSN剪切)与DNA探针杂交后,DSN对DNA探针进行剪切,被释放的启动子经过不断地循环扩增后,可产生大量的不同长度的带有荧光基团的DNA裂解探针,实现了检测信号的扩增;(3)信号的分离主要由DNA探针的碱基序列和长度控制,其出峰时间和前后顺序均可优化调整,提高了信号的分离效率。

## 1 实验部分

### 1.1 仪器、试剂与材料

本研究中使用的所有核酸均由上海生工生物科技有限公司合成并通过HPLC纯化,它们各自的序列在附表S1(详见https://www.chrom-China.com)中列出。MBs-Dynabeads(链霉亲和素包被的M-300)购自无锡百迈格生物科技有限公司。DSN(50 U)购自Evrogen公司(莫斯科,俄罗斯)。Tween 20由上海安耐吉化学提供。三(羟甲基)氨基甲烷(Tris)、氯化镁(MgCl_2_)、氯化钠(NaCl)、乙二胺四乙酸(EDTA)、HPLC级乙腈和甲醇均购自中国上海医药集团公司。醋酸三乙铵(TEAA)缓冲液购自Sigma-Aldrich公司(密苏里州圣路易斯,美国)。组胺、色胺、精胺和酪胺均购自中国上海麦克林生化科技有限公司。抗生素来自江苏省食品药品监督管理局。附表S2中列出了本工作中使用的缓冲液的组成,包括偶联缓冲液、洗涤缓冲液以及杂交缓冲液。所有溶液均使用无核酸酶的超纯水制备,该设备购自深圳亿利源水处理设备有限公司,其电阻为18.2 MΩ·cm。

离心分离使用Sigma 3-15离心机(哈尔茨奥斯特罗德,德国)进行。pH值测量使用中国上海三鑫电子设备有限公司的带有玻璃-甘汞电极的SX-610数值pH计进行。荧光测量在FS5分光光度计(英国爱丁堡仪器,柯克顿校区)上进行。

### 1.2 实验条件

#### 1.2.1 长、短探针偶联物的制备

根据之前的工作^[19]^,将被生物素标记的适配体(Aptamer 1、Aptamer 2、Aptamer 3和Aptamer 4)偶联至链霉亲和素涂层的磁珠表面。首先,取30 μL磁珠加入到2 mL离心管中,离心90 s后,放置在磁力架上90 s。随后弃去溶剂并将磁珠保留在离心管中。然后使用偶联缓冲液低档振荡清洗3次后,同样放置在磁力架上90 s,弃去溶剂保留磁珠。将磁珠重新悬浮于洗涤缓冲液中,同时加入4种不同的适配体,在室温下轻轻涡旋一段时间。涡旋结束后,将得到的适配体-磁珠偶联物在4 ℃下保存备用。为了选择最佳的偶联时间以及估算DNA探针在磁珠上的偶联效率(IE)和百分含量(IP)。将3'端被荧光素标记的适配体加入到经过上述同样洗涤磁珠步骤后的离心管中,在室温下轻轻涡旋不同的时间,并将其置于磁力架上90 s。偶联时间为30 min。随后,将被生物素标记的探针(Probe 1、Probe 2、Probe 3和Probe 4)偶联至链霉亲和素涂层的磁珠表面,这与适配体偶联至磁珠的步骤相同。在含有磁珠的洗涤缓冲液中,同时加入4种不同的长、短探针,在室温下轻轻涡旋一段时间。偶联时间为25 min。

#### 1.2.2 生物胺的检测

将60 μL的Aptamer 1、Aptamer 2、Aptamer 3、Aptamer 4与磁珠的偶联物加入到离心管中,然后依次加入15 μL 1 μmol/L的RNA启动子(Trigger 1、Trigger 2、Trigger 3、Trigger 4)和80 μL反应缓冲液(50 mmol/L Tris-HCl, pH 7.0, 30 mmol/L MgCl_2_),在37 ℃下反应1 h。然后依次将15 μL 1 μmol/L的酪胺、组胺、精胺和色胺在37 ℃下反应2 h。反应结束后,将其置于磁力架上90 s,取上清液加入到含有60 μL Probe 1、Probe 2、Probe 3、Probe 4与磁珠偶联物的棕色离心管中。然后加入0.9 U DSN和60 μL反应缓冲液。将反应混合物涡旋振荡10 s以充分混合。在40 ℃下孵育210 min后,用5 μL DSN终止液停止DSN对DNA链的裂解。将其置于磁力架上90 s,取上清液注入HPLC中进行检测。

#### 1.2.3 HPLC方法

使用悟空K2025系统对样品进行HPLC-荧光检测,该系统带有高压输液泵、自动进样器、柱温箱和RF-20A荧光检测器。数据处理使用LCsolution软件。全多孔杂化硅胶柱Phenomenex Clarity^®^3 μm Oligo-RP色谱柱(50 mm×4.6 mm, 3 μm)用于多种核酸的分离。相比于传统的硅胶色谱柱,Oligo-RP更适合对核酸的分离分析。柱温设置为35 ℃。采用含有5%(v/v)乙腈的醋酸三乙胺(100 mmol/L, TEAA, pH=7.0)溶液(A)和甲醇(B)为流动相,流速为1.0 mL/min。梯度洗脱模式为0~20 min, 10%B~20%B; 20~25 min, 20%B~10%B; 25~30 min, 10%B。荧光检测器的激发波长和发射波长分别设置为488 nm和520 nm。

#### 1.2.4 实际样品的前处理

取1 g肉类样品加入到含有10 mL 1.0 mol/L HCl的离心管中,7750 r/min离心20 min。随后取2 mL上层清液加入到15 mL的离心管中,向离心管中依次加入0.13 g NaCl、2 mL 2.5 g/L丹磺酰氯溶液以及0.8 mL 6.0 mol/L NaOH,混合均匀后,衍生化反应5 min,随后以7750 r/min离心1 min,离心后将上层溶液收集在色谱样品瓶中,在4 ℃下储存备用。

## 2 结果与讨论

### 2.1 实验条件考察

#### 2.1.1 方法机理

图1为基于适配体置换生物胺信号源并结合DSN辅助的目标循环扩增策略同时检测4种生物胺(酪胺、组胺、精胺、色胺)的原理图。如图1所示,首先使用生物胺核酸适配体标记磁珠,并与Trigger结合组装双链结构。在检测过程中,生物胺与适配体结合并释放启动子,使其与修饰有荧光基团的不同长度的DNA核酸探针分别结合,形成DNA-RNA异源双链。加入的DSN可以特异性地裂解异源双链DNA,同时保持RNA完整性。然后,释放的启动子重新参与下一轮的循环。在足够长的孵育时间内,经过不断地杂交、裂解和释放,可以得到大量的具有荧光信号的DNA裂解探针。由于碱基长度和序列的差异,被剪切的DNA探针在HPLC中的保留时间不同。因此,代表不同生物胺的DNA裂解探针可以在HPLC中完美分离,从而实现在一次运行中对4种生物胺的定量检测。

**图1:**
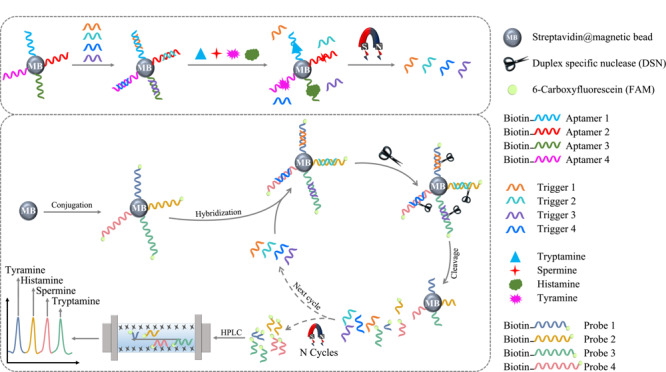
核酸适配体置换信号结合循环扩增对4种生物胺检测的示意图

#### 2.1.2 不同碱基序列的长、短荧光探针在HPLC中的保留时间探究

研究发现,寡核苷酸的碱基序列和长度影响了它们在色谱中的保留时间。在单链核酸中,碱基的疏水性顺序为胞嘧啶(C)<鸟嘌呤(G)<腺嘌呤(A)<胸腺嘧啶(T)。疏水性越低,在反相色谱中的保留越弱,出峰时间越早^[20]^。一般来说,寡核苷酸的保留强弱取决于碱基与流动相、固定相之间的疏水作用。但碱基的疏水性太高,即使在核酸专用的Oligo-RP色谱柱中,它们的保留也非常弱。为了增加寡核苷酸在色谱柱上的保留时间,在流动相中加入了TEAA离子对试剂。TEAA既带正电荷又具有疏水性,因此不仅TEAA的正电荷能与核酸主链上的磷酸基团的负电荷反应,同时TEAA的疏水基团又与固定相上十八烷基的疏水基团发生反应。这种离子对试剂起着连接核酸和柱基质之间的桥梁作用,使DNA片段吸附在固定相上面。

通过将DNA探针设计成具有不同的碱基序列和长度的核酸链,研究了其在HPLC中的保留时间(见图2)。将色谱条件的梯度洗脱模式设置为20 min内甲醇的比例由10%增加到30%。可以看出,当核酸链中的碱基个数相同时,C、A和T的保留顺序为C_4_<A_4_<T_4_。对于C和A来说,它们和离子对之间的静电作用随着核酸长度的增加而增大,因此得到的离子对与固定相之间的疏水相互作用也随着C和A碱基个数的增加而增加,因此随着C和A核酸链长度的增加,它们的保留时间增加。考虑到分离效率和实验成本,最终选择了C_4_、C_12_、C_30_和T_4_作为DNA探针的尾部,其保留时间依次增加。

**图2:**
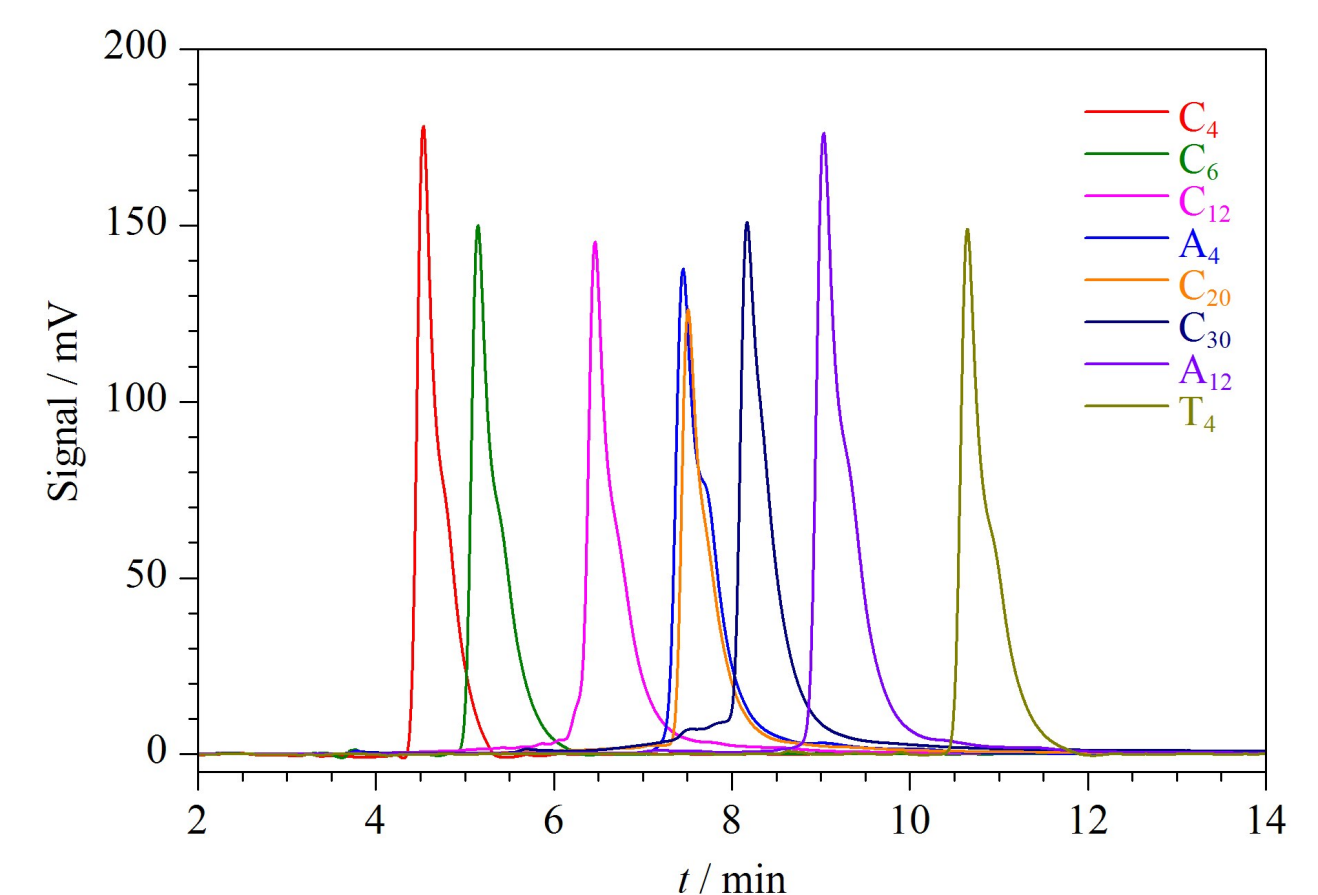
不同碱基序列和长度的核酸链在HPLC中的保留

#### 2.1.3 HPLC方法的优化

为了提高C_4_、C_12_、C_30_和T_4_ 4个色谱峰之间的分离效率,需要选择合适的HPLC条件进行优化。柱温、流速和梯度洗脱过程是影响色谱峰分离度的3个主要因素。在梯度洗脱条件中包括梯度洗脱时间和梯度洗脱比例两个方面。在此,将C_4_、C_12_、C_30_和T_4_等浓度一同进样,分别改变梯度洗脱时间和梯度洗脱比例,探究这两个方面对分离度的影响。结果表明,改变梯度比例对分离度的影响较大(见附图S1),因此在正交试验中选择梯度比例作为优化的一个因素。因此以梯度比例、柱温和流速3个因素分别设置3个水平进行正交试验,可以分别得到*R*_1_、*R*_2_和*R*_3_ 3个分离度(见附表S3~S4)。然后分别对*R*_1_、*R*_2_、*R*_3_进行极差分析和方差分析(见附表S5~S8),通过对各因素水平间的多重比较(见附表S9~附表S17),分别得到对*R*_1_、*R*_2_、*R*_3_来说最佳的实验组合。对*R*_1_、*R*_2_和*R*_3_来说,最佳的色谱条件均为在35 ℃的柱温下,甲醇以1.0 mL/min的流速在0 ~ 20 min内从10%变化到20%(见附图S2)。因此最终选择1.2.3节色谱条件作为本实验的HPLC分析方法。在优化后的色谱条件下,C_4_、C_12_、C_30_和T_4_的色谱图如图3所示,色谱峰的分离度分别是3.442、3.591和2.371,实现了4个峰之间的完全分离。

**图3:**
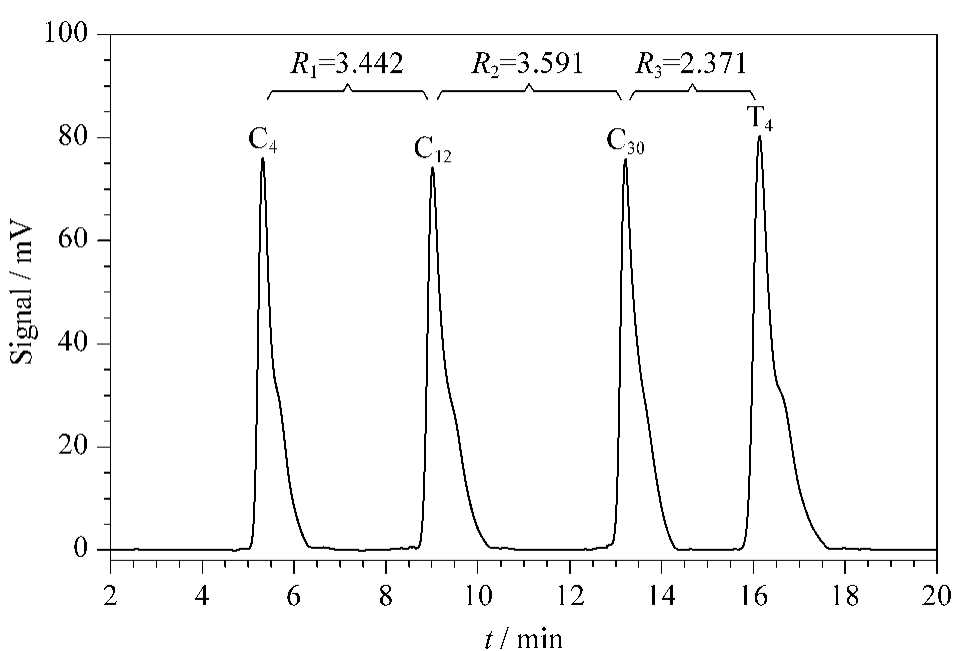
正交试验优化后4种DNA探针的色谱图

#### 2.1.4 其他条件优化

进一步优化了实验条件(包括生物胺与适配体的孵育时间、DSN用量、Mg^2+^浓度、缓冲液的pH值、孵育温度和DSN孵育时间),将生物胺的浓度设定为1 μmol/L,适配体和启动子的浓度都保持在1 μmol/L,探针的浓度为100 μmol/L,优化结果见附图S3。最终选择的实验条件为:生物胺与适配体孵育时间120 min; DSN用量0.90 U; Mg^2+^浓度30 mmol/L; pH值7.0;孵育温度40 ℃; DSN孵育时间210 min。

### 2.2 方法学考察

#### 2.2.1 可行性分析

为了验证这种方法的可行性,在相同的反应条件下,对不同样品进行有/无生物胺或DSN的检测结果进行对比,如图4所示。在没有目标生物胺的情况下,无论是否加入DSN,几乎都没有色谱峰出现(图4a和4b)。当仅有生物胺、适配体、启动子和探针存在的时候,虽然适配体和探针能够形成DNA-RNA异源双链,但没有DSN酶对其DNA链进行切割,进而无法产生裂解的DNA探针。因此也几乎不会出现目标物对应的色谱峰(图4c)。只有当目标生物胺和DSN同时存在时,如图4d、e、f和g所示,才会有相对应的信号出现。当4种生物胺和DSN都存在时,可以观察到这4种目标物对应的色谱峰的峰面积显著增加(图4h)。以上结果表明,该方法可以在一次进样中实现对酪胺、组胺、精胺和色胺的高灵敏检测。

**图4:**
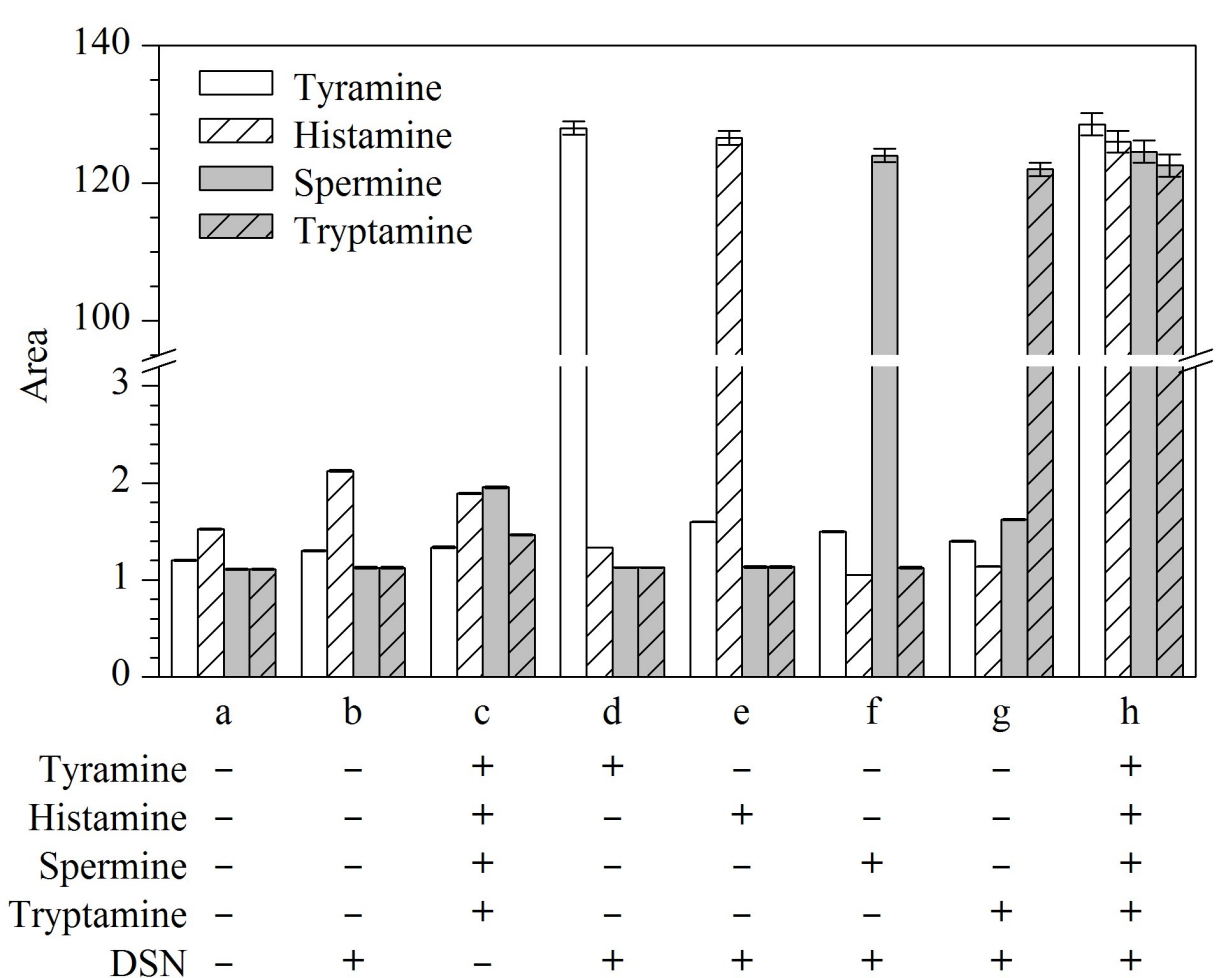
可行性分析(*n*=3)

#### 2.2.2 检测性能的分析

为了评价该方法对生物胺的检测性能,在最佳的反应条件下,对不同浓度的生物胺所产生的信号响应进行了研究(见图5a)。酪胺、组胺、精胺和色胺对应的DNA裂解探针的保留时间分别为5.69、9.26、13.40和16.10 min。为了评估这4种峰的分离度,计算了生物胺在1 μmol/L时所对应的色谱峰的分离度,如图5b所示。其中*R*_1_为3.06, *R*_2_为3.62, *R*_3_为2.19。分离度均大于1.5,说明4个峰已完全分离。在1 pmol/L的浓度下,4种目标物所对应的色谱峰仍然可以被明显地分离。

**图5:**
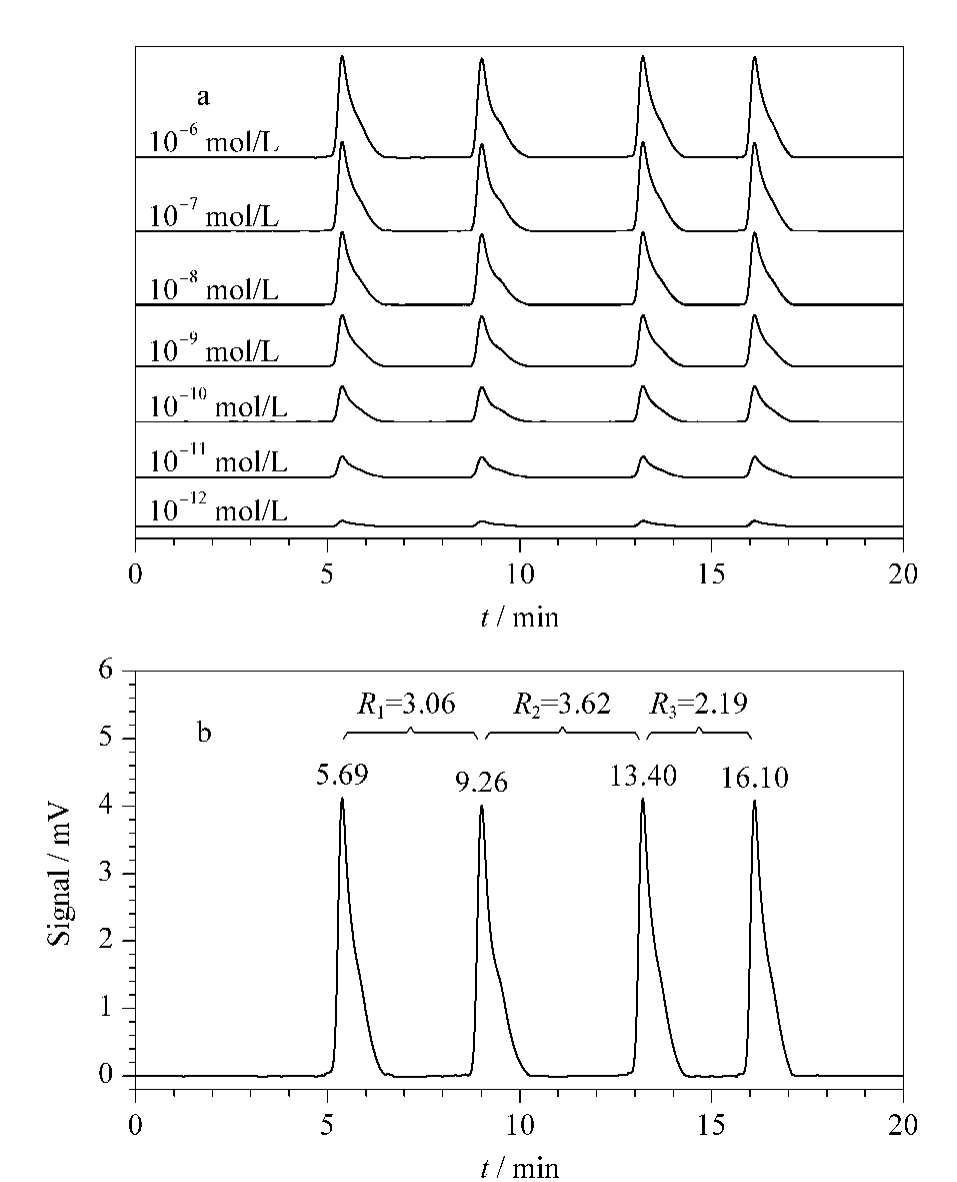
(a)不同浓度下目标物的色谱图和(b)1 μmol/L生物胺对应的4个色谱峰的分离度

不同浓度生物胺检测的线性关系如附图S4所示,在1 pmol/L~1 μmol/L范围内,峰面积与生物胺浓度的对数呈良好的线性关系,决定系数(*R*^2^)分别为0.995、0.992、0.991、0.995。酪胺、组胺、精胺和色胺的LOD分别为0.25、0.21、0.27和0.19 pmol/L。在整个线性范围内,经过3次重复测量,RSD均小于5%,充分表明了检测方法的准确性。

### 2.3 实际样品检测

为了评估所建立方法的实用性,对从超市买来的新鲜草鱼和猪肉进行了检测,结果如表1所示,在鱼肉中检测到了12.321 nmol/L的酪胺和14.676 nmol/L的组胺,在猪肉中检测到了16.982 nmol/L的组胺、17.756 nmol/L的精胺和13.172 nmol/L的色胺。为了评估基质效应,向预处理得到的鱼肉和猪肉样品溶液中分别加入了1 nmol/L和10 nmol/L的酪胺、组胺、精胺和色胺标准溶液,得到的相对回收率在101.2%~104.5%之间,本方法的相对标准偏差(RSD)在1.5%~4.3%之间。鱼肉和猪肉中加标前后的色谱图见附图S5。为了与传统方法进行比较,直接通过HPLC-紫外检测器对鱼肉中存在的酪胺和组胺进行检测。标准曲线如附图S6a所示,其*R*^2^均是0.997。在该传统的方法中,酪胺和组胺的LOD分别是15.23 nmol/L和15.07 nmol/L。随后我们用注射器取1 g已经放置了3 d的鱼肉,经过衍生化之后,分别使用本论文的方法和传统的HPLC-紫外检测器直接检测法对生物胺含量进行检测,并将检测结果进行对比。根据我们先前的研究^[21]^,肉类样品在贮藏过程中,生物胺的含量会急剧上升,3 d后即可达到较高水平。因此,这里选择放置3 d的鱼肉进行检测,以更清晰直观地将本文方法与传统方法进行比较。结果如附表S18所示,本方法和传统的HPLC方法结果基本吻合。附图S6中的b图和c图分别是传统HPLC方法和本方法加标前后的鱼肉样本的色谱图。以上结果均能说明,该方法对实际样品中多种生物胺的检测具有良好的实用性。我们用同样的方法检测了硫酸庆大霉素中的生物胺,从表1的结果中可以看出,在硫酸庆大霉素中可以检测到精胺的存在。表2列出了一些常见的检测生物胺的方法,并比较了它们之间的检测性能。从表2可以看出,我们的方法具有更低的LOD和更宽的线性范围,充分表明了该方法优越的检测性能。

**表 1:** 检测新鲜鱼肉、猪肉以及硫酸大庆霉素中的生物胺

Sample	Spiked/ (nmol/L)	Tyramine		Histamine		Spermine		Tryptamine	
		Found/ (nmol/L)	Relative recovery/%	Found/ (nmol/L)	Relative recovery/%	Found/ (nmol/L)	Relative recovery/%	Found/ (nmol/L)	Relative recovery/%
Fish meat	blank	12.321	-	14.676	-	ND	-	ND	-
	1	13.356	103.5	15.711	103.5	1.034	103.4	1.027	102.7
	10	22.572	102.5	24.793	101.2	10.295	103.0	10.453	104.5
Pork	blank	ND	-	16.982	-	17.756	-	13.172	-
	1	1.021	102.1	17.997	101.5	18.769	101.3	14.196	102.4
	10	10.432	104.3	27.234	102.5	28.122	103.7	23.511	103.4
Gentamycin	blank	ND	-	ND	-	0.026	-	ND	-
sulfate	1	1.017	101.7	1.041	104.1	1.052	102.6	1.036	103.6
	10	10.34	103.4	10.26	102.6	10.166	101.4	10.12	101.2

ND: not detected; -: no data.

**表2:** 多种生物胺检测策略的比较

Detection technique	Targets	Samples	LODs/(pmol/L)	Linear range/(pmol/L)	Ref.
HPLC-UV	histamine	fish	5×10^6^-5×10^7^	0-2×10^8^	[22]
Chemiluminescence	histamine	fish, pork	3.2×10^3^	1×10^5^-1×10^8^	[23]
Colorimetric analysis	putrescine	fish	1.9×10^7^-6.4×10^7^	6×10^8^-2×10^11^	[24]
HPLC-fluorometry combine nucleic	tyramine, histamine,	fish, pork	0.25, 0.21, 0.27, 0.19	1-1×10^5^	this work
acid amplification strategy	spermine, tryptamine				

## 3 结论

综上所述,本文开发了一种基于适配体置换出生物胺信号源并且结合DSN辅助目标物循环扩增的HPLC检测策略,并成功用于发酵类抗生素和肉类样品中多种生物胺的同时识别和高灵敏检测。适配体与DSN辅助启动子循环扩增策略的结合有效提高了对目标的选择性和灵敏度。探针的碱基序列种类和长度的控制、影响因素的正交分析以及实验条件的优化大大提高了目标物的色谱分离度。重要的是,多种生物胺能够通过一次进样就得以识别,这可以有效弥补传统色谱检测方法的一些不足。该方法可扩展至其他食品药品有害物的同时检测,在食品药品的高灵敏分析领域具有一定的实际应用价值和潜在的开发前景。
